# A Comparison of the Ligation Torque Expression of a Ribbonwise Bracket–Archwire Combination and a Conventional Combination: A Primary Study

**DOI:** 10.1155/2022/9251172

**Published:** 2022-09-28

**Authors:** Bin Lin, Feifei Jiang, Jie Chen, Jiaxing Liang

**Affiliations:** ^1^Department of Orthodontics, Fujian Medical University Union Hospital, Fuzhou, Fujian Province, China; ^2^Shenzhen Institute of Advanced Technology (SIAT), Chinese Academy of Sciences (CAS), Shenzhen, Guangdong Province, China; ^3^Department of Mechanical and Energy Engineering, Indiana University Purdue University Indianapolis, Indianapolis, IN, USA; ^4^Department of Orthodontics and Oral Facial Genetics, Indiana University, Indianapolis, IN, USA

## Abstract

**Objective:**

To assess the effect of the third-order mechanics of a new ribbonwise bracket–archwire combination using an orthodontic torque simulator. *Material and Methods*. An orthodontic torque simulator was used to measure the third-order moment of a maxillary central incisor as it changed from a neutral position to a 40° rotation in 1° increment. A new ribbonwise bracket (Xinya, China) was compared with a conventional ligation bracket (American Orthodontic, U.S.A.). The effects of different archwire sizes (i.e., 0.017″ × 0.025″ and 0.019″ × 0.025″) and materials (i.e., nickel-titanium, titanium-molybdenum alloy, and stainless steel) were analyzed. Paired sample *t*-tests were conducted to compare the moments between the two bracket types corresponding to each of the archwires. The effects of the stiffness of the bracket–archwire complexes were also assessed.

**Results:**

Statistically significant differences (*P*=0.05) between the moments from the two brackets were found. The ribbonwise bracket–archwire complex generated larger moments when the rotation angle was lower than 30°. The ribbonwise brackets produced moments that could reach a threshold of 5 Nmm more quickly as the angle was increased. The higher the stiffness of the complex, the larger the moment.

**Conclusion:**

The ribbonwise bracket–archwire complex reached the moment threshold limits earlier than the conventional complex. When the rotation angle is less than 30°, the ribbonwise bracket–archwire complex generated a greater torque moment in comparison with the conventional complex.

## 1. Introduction

Orthodontic torque refers to labio-lingual root tipping, in which the movement of the crown is minimized and the movement of the root apex is maximized [1]. With contemporary fixed appliances, the labio-lingual inclination can be corrected through the third-order moment generated by twisting a rectangular wire in the bracket slot, as described by Rauch [2]. For a conventional bracket, although the moment can be adjusted by filling the bracket slot and gradually increasing the archwire dimensions [3], a considerable percentage of the moment is lost due to the play between the bracket slot and archwires [4, 5].

The third-order moment needs to be controlled within a certain range to achieve the desired effect. The moment should not exceed a certain limit to avoid clinical side effects [6, 7]. Although there is no scientific consensus regarding the ideal moment, most scholars agree that in clinical practice, the values of effective moments range between 10 and 20 Nmm [8, 9], and several have suggested values of 5–20 Nmm [10, 11]. The minimum torque required for a maxillary incisor has been reported to be 5 Nmm [12].

**Figure 1:**
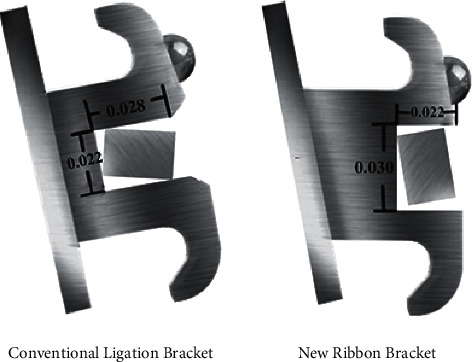
new ribbonwise labial bracket contains an occlusoapical slot of 0.030″ × 0.022″, in contrast with the conventional ligation bracket which is 0.022″ × 0.028″. The wire is 0.017″ × 0.025″ as a contrast.

In recent years, a new ribbonwise labial bracket (Figure 1) has been introduced clinically. Its 0.030″ × 0.022″ slot is wider in the occlusal-apical direction than in the lingual-labial direction, while a conventional bracket has a 0.022″ × 0.028″ slot, with a wider lingual-labial side than occlusal-apical side. The ribbonwise arch appliance is the first orthodontic appliance with the capacity for three-dimensional control of tooth movement [13]. Similar ribbonwise brackets have been incorporated into lingual brackets [14]. A previous study aimed at a specific clinical case indicates that lingual brackets with a wider occlusal-apical slot generate higher torques than conventional brackets with a wider lingual-labial slot [15]. However, the general behavior of ribbonwise brackets has not been experimentally studied.

**Figure 2:**
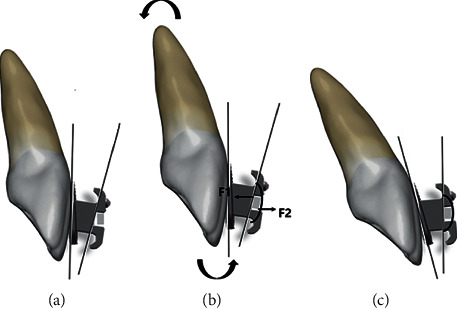
Schematic of the torsion application system. Sagittal view of new ribbonwise bracket during its realization of “ligation torque expression.” (a) No torque will be transmitted when any archwires are placed vertically in the 0.030″ × 0.022″ slot since the slot is much larger. (b) The steel ligatures were tightened as tight as possible thus the archwire was securely pressed onto the slot bottom. (c) Torque will be derived from the contact of the slot bottom and the ligature wire, then torque will be expressed in the arrow direction.

The ribbonwise bracket is used with a new method of torque expression called ligation torque expression, which involves tying the ribbonwise archwire tightly using a stainless-steel ligature wire to ensure it conforms to the bottom of the bracket slot to reduce the moment due to undesired play (Figure 2). Compared with traditional brackets, the moment is generated by the ligature wire's pre-deformation rather than the bracket slot.

Although the new ribbonwise bracket design leads to better torque control because the corresponding ligation method eliminates the play between the ribbonwise bracket and archwire, its behavior as a function of the design parameters, e.g., the wire size, bracket size, and wire materials, has yet to be elaborated. It is imperative to ensure that the new design can produce the desired moment (5 Nmm) and determine whether it performs better than conventional brackets.

The objectives of this study are to (1) quantify the moments generated by the third-order mechanics of the new ribbonwise bracket–archwire complex with different types of archwires, (2) identify the advantages of ribbonwise and conventional brackets, and (3) determine the parameters that control the moment.

## 2. Materials and Methods

### 2.1. Experimental Apparatus

**Figure 3:**
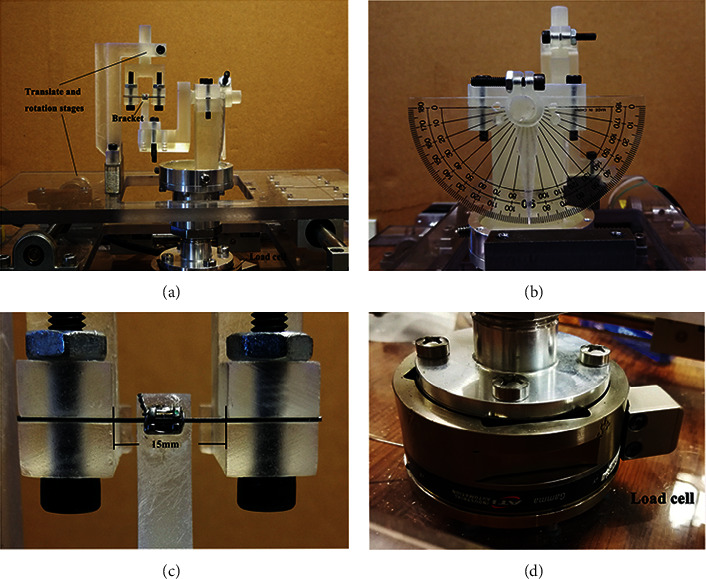
The torque measurement device.

An orthodontic torque simulator developed by the Department of Mechanical and Energy Engineering of Indiana University-Purdue University Indianapolis (Figure 3(a)) was used to measure the third-order moment on a maxillary central incisor generated by a bracket–archwire complex. It consisted of a platform, wire-fixing clamps, a six-axis load cell (Multi-axis Force/Torque Nano17, ATI Industrial Automation, Apex, NC), and an artificial central incisor model. The tooth could be rotated around the mesial-distal axis to simulate the twisting of the archwire and could be locked at any angle to simulate different applied torques (Figure 3(b)). A straight archwire was fixed to the wire-fixing clamps, with the midpoint located at the center of the bracket slot (Figure 3(c)). A six-component force/moment load cell (Figure 3(d)) was connected to the tooth directly to measure the forces and moments. After the wires were dropped into the bracket slot at the initially estimated zero position, by adjusting the adjustable stages and rotatable bracket dowel, initial loads were zeroed within to 0.01 N and 0.05 Nmm for forces and moments, respectively, in all directions, to achieve three-dimensional alignment of the slot of the bracket and the tested archwire and neutralize preexisting angles of each bracket. The distance between the wire-fixing clamps was determined to be 15 mm (Figure 3(c)), which was the average distance between the brackets on the neighboring teeth [16].

### 2.2. Study Materials

**Figure 4:**
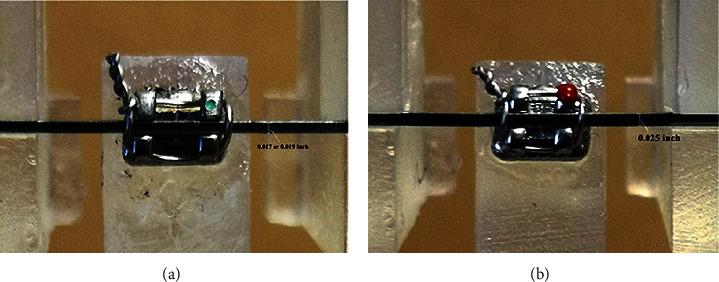
The conventional strength wire bracket (a) and the new ribbonwise bracket (b) are ligated in the OTS for testing using the experimental configuration.

Two types of brackets, i.e., the new ribbonwise bracket (Xinya, Hangzhou, China) and the conventional ligation bracket (American orthodontic, Sheboygan, Wisconsin, USA), were compared. The left maxillary central incisor brackets were chosen for the test. The ribbonwise bracket had a 0.030″ × 0.022″ slot that was wider in the occlusal-apical direction than the lingual-labial direction to contrast with the conventional ligation bracket, which had a 0.022″ × 0.028″ slot that was wider in the lingual-labial direction. Six different types of archwires were used for each bracket, as follows: two differently sized archwires at 0.017″ × 0.025″ and 0.019″ × 0.025″ made of either nickel-titanium (NiTi) (IMD Medical Inc., Shanghai, China), titanium-molybdenum alloy (TMA) (Ormco, Glendora, California, U.S.A.), or stainless steel (SS) (Ormco, Glendora, California, U.S.A.). The archwires ligated into the ribbonwise bracket were adjusted to be placed ribbonwise (Figure 1). All archwires were ligated into the bracket slot using a 0.012″ ligature wire (Ormco, Glendora, California, U.S.A.). There were 12 sample groups of 10 specimens each for a total of 120 bracket-wire specimens (Table 1, Figure 4).

Third-order rotations were introduced by rotating the bracket from a neutral position up to a 40° rotation at 1° increment (Figure 1). The 10 specimens in each bracket–archwire complex group were tested over the entire range of rotation.

The SS ligatures were firmly tightened; thus, the archwire was securely pressed onto the slot bottom to ensure the initial seating of the archwire with consistent and similar ligation forces [17]. All ligations and measurements were performed by the same investigator, who had been practicing orthodontics for 14 years. A three-minute waiting period was allocated to allow a reproducible amount of stress relaxation to occur [18].

The six-axis load cell (Figure 3) measured the forces and moments at the bracket; this method has been reported previously [19]. The torque and angle prescribed in the brackets did not affect the current study because the neutral position of zero torque was used as the baseline.

### 2.3. Statistical Analysis

The mean and standard deviation of the generated moments from the 10 repeated measurements for each group were calculated. Significance was defined as *P*=0.05. Paired sample *t*-tests were carried out for the comparison of the moments between two types of brackets with one type of archwire separately. To determine whether the curves were linearly related and evaluate the slope (moment/angle) of each bracket–archwire combination, the linear regression method was used to establish the linear equation according to the linear section of the moment-angle curve [20]. All statistical analyses were performed with SPSS (version 20.0, SPSS, Chicago, Ill).

## 3. Results

**Figure 5:**
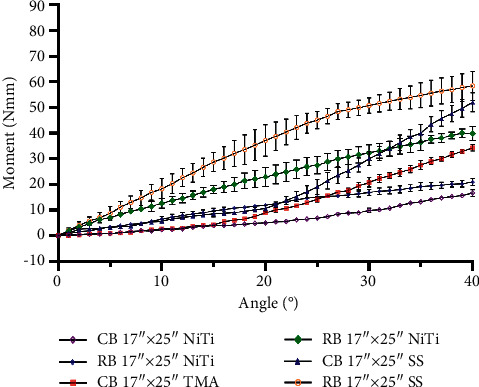
Measured moments acting on the incisor with combinations of brackets and 0.017″ × 0.025″ archwires.

**Figure 6:**
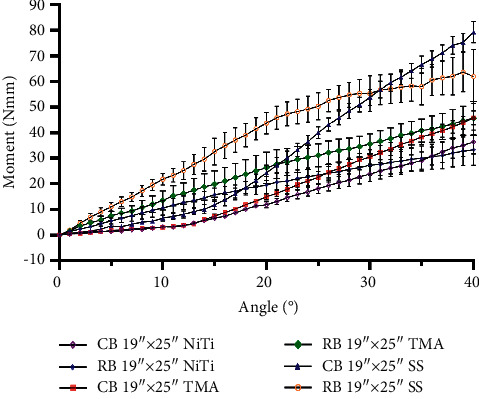
Measured moments acting on the incisor with combinations of brackets and 0.019″ × 0.025″ archwires.

The moments generated by each bracket type are shown in Table 2, and the moment-angle curves are depicted in Figures 5 and 6. The curves had smaller slopes before the angle reached the play angle. The ribbonwise bracket showed completely linear trends, except for when SS was used, which led to a loss of linearity upon reaching 27° for the 0.017″ × 0.025″ archwire and 22° for the 0.019″ × 0.025″ archwire. The ribbonwise bracket generated higher moments in most cases, except for the 19″ × 25″ SS and NiTi archwires when the angle exceeded 35°. Its slope was larger than that of the first section of the conventional bracket but smaller than that of the conventional bracket's second section. The slopes were evaluated by linear regression within the linear part of the curve (Table 3).

The wire stiffness affected the moment level. The stiffer the wire, the larger the moment (Table 2). For the conventional bracket and 0.017″ × 0.025″ archwire, the maximum moment at 40° was 17, 34, and 52 Nmm for the NiTi, TMA, and SS materials, respectively; for the ribbonwise bracket, the maximum moment at 40° was 21, 40, and 58 Nmm for the NiTi, TMA, and SS materials, respectively. For the conventional bracket and 0.019″ × 0.025″ archwire, the maximum moment at 40° was 36, 46, and 79 Nmm for the NiTi, TMA, and SS materials, respectively; for the ribbonwise bracket, the maximum moment at 40° was 33, 46, and 62 Nmm for the NiTi, TMA, and SS materials, respectively.

The ribbonwise bracket-wire complex reached the moment thresholds of 5 and 20 Nmm earlier than the conventional complex. The angles at which the torque fell between the clinical threshold of 5.0 Nmm and the recommended maximum limit of 20 Nmm for each bracket–archwire combination are reported in Table 4. Regarding reaching the 5 Nmm thresholds using the 0.017″ × 0.025″ archwire, the conventional bracket surpassed this level between 10° and 20° of archwire rotation, while the ribbonwise bracket reached this limit in the 3°–9° degree range. For the 0.019″ × 0.025″ archwire, the conventional bracket reached the two limits in the range of 8°–14° of rotation, while the ribbonwise bracket reached the limits in the range of only 3°–6°.

## 4. Discussion

The conventional bracket-wire complex showed the piecewise linear moment-angle curves due to the play, similar to previously reported results [21], while the ribbonwise bracket-wire complex showed linear trends with a moderate stiffness within the clinically desired range, as shown in Figures 5 and 6. The study results indicated that the ribbonwise bracket reached the threshold of 5 Nmm earlier at 3°–6° (except when using the 0.017″ × 0.025″ NiTi wire) versus the conventional bracket, which reached the threshold at 8°–14°. In general, the linearity of the moment and angle of the ribbonwise bracket was better than that of the conventional bracket.

The new ribbonwise bracket–archwire complex provided enough torque for all six types of archwires. All bracket-wire complexes reached 20 Nmm, except for the 0.017″ × 0.025″ NiTi wire. The ribbonwise bracket exceeded the upper limit sooner when the angle was gradually increased (Figures 5 and 6). The moment-angle relationship of the SS archwire at a high angle was not linear, possibly due to plastic deformation of the ligation wire when the moment exceeded its elastic limit.

The archwire stiffness also played a significant role in the level of moment generated. The higher the stiffness, the larger the moment generated under the same rotating angle. The stiffness could be changed using different archwire sizes and materials (Figures 5 and 6).

The slot-wire play in the conventional bracket could influence the transference of the ideal torque prescribed in the bracket. Under all conditions, the wires in the conventional bracket produced the moment in a piecewise linear pattern. The curve showed a linear pattern with a smaller slope before the archwire was fully engaged. However, while the stiffness of the conventional bracket-wire complex was represented by the slope of the moment-angle curve, the stiffness of the ribbonwise bracket-wire complex was lower when fully engaged. Table 3 shows the slope representing the torque-angle ratio. With the conventional bracket, the moment was produced by the unstable ligature in the first section (the ligature stiffness) and the twisting of the rectangular wire against the walls of the rectangular bracket slot in the second section (the binding stiffness). The stiffness of the ribbonwise bracket accounted for 64%–88% of the binding stiffness of the conventional bracket. In course of orthogonal tool movement, as long as the deformation of the archwire reaches the effective torque, the torque could be effectively expressed. The rectangular archwire has good flexibility on the wide surface, so it is not difficult to deform the archwire by ligation to achieve effective torque. In the treatment, the ribbonwise archwire is tightly tied to the bottom of the bracket slot with the ligation wire, which causes the deformation of the archwire to reach the effective torque, and the tooth will have torque movement. With the movement of the teeth and the resilience of the archwire, the archwire and the bottom of the bracket slot will gradually fully fit. The original preset angle on the bracket will be reflected on the teeth. The ribbonwise bracket-wire complex showed linear trends while the torque magnitude increased as the angle increased starting from 0°. It indicated that there was no play and that the moment produced by the archwire was translated to the bracket immediately. For this reason, the maximum moment of the ribbonwise bracket-wire complex produced at 40° was not smaller than that of the conventional one, although its slope was lower. The ribbonwise bracket could effectively increase the moment by minimizing the effect of play that occurred when using the conventional bracket.

For conventional brackets with a 0.022-inch bracket slot, stainless steel archwires of 0.021 inches, which are very similar in size to the slot, are so limited in springiness and range of torsion that effective torque with the archwires is impossible. The use of NiTi wires, TMA wires, torque auxiliaries, smaller rectangular steel wires, or exaggerated inclinations has been reported to overcome this limitation [22, 23]. However, these factors also increase the complexity of clinical practice. By contrast, the new ribbonwise bracket only requires firmly tying the ligation wire, which is an easier method for achieving the desired moment. The purpose of ribbonwise brackets should be tested in future investigations, in order to understand the behavior of this technique in combination with other bracket materials, such as ceramic [24] and fiber-reinforced composite [25]. Furthermore, this study quantitatively characterized the moment-generating behavior of the proposed ribbonwise bracket. However, the conclusions are qualitative due to discrepancies between the experimental setting and actual clinical practice, which may result in different clinical responses. The anisotropic periodontal ligament, tooth morphology, especially crown morphology, individual responses to moments applied, variability in malocclusion, and saliva may result in a variety of clinical responses to a given bracket–archwire complex [26]. The findings of this research provided insight into future bracket design based on the Angle ribbon bracket.

This research also has limitations. Present results can only be used as elementary evidence for clinical work by comparing the differences in the torque mechanisms of the two brackets. Relevant research on other aspects of the new ribbonwise bracket system, such as the long-term effect and stability of the ligation, instead of a single bracket experiment, an original aligned dental arch which is applied to simulate the clinical dental arch state, must be carried out.

## 5. Conclusion

1. The ribbonwise bracket reached the threshold limits of 5 and 20 Nmm earlier than the conventional bracket.
2. The ribbonwise bracket–archwire complex generated a greater torque moment when the rotation angle is less than 30° in comparison with the conventional complex.
3. The archwire stiffness played an important role in the level of moment generated. The higher the stiffness, the larger the moment.
4. The stiffness of the ribbonwise bracket fell between the ligature stiffness and binding stiffness of the conventional bracket.

## Figures and Tables

**Table 1:** Brackets and archwires were used in this study.

Bracket	Manufacturer (abbreviation)
Conventional bracket	American orthodontic, Sheboygan, Wisconsin, USA (CB)
New ribbon bracket	Xinya, Hangzhou, China (RB)

*Archwire*	
0.017 × 0.025 inch NiTi	IMD Medical inc, Shanghai, China
0.017 × 0.025 inch TMA	Ormco, Glendora, California, USA
0.017 × 0.025 inch SS	Ormco, Glendora, California, USA
0.019 × 0.025 inch NiTi	IMD Medical inc, Shanghai, China
0.019 × 0.025 inch TMA	Ormco, Glendora, California, USA
0.019 × 0.025 inch SS	Ormco, Glendora, California, USA

NiTi indicates nickel titanium; TMA indicates titanium molybdenum alloy; SS indicates stainless steel.

**Table 2:** Mean, standard deviation (SD), mean difference, and *t*-test result of the moment (Nmm) at every 5 degrees for different experimental configurations (*n* = 10).

Archwires	Degree of rotation(°)	Conventional bracket		New ribbon bracket			
		Mean (Nmm)	SD	Mean (Nmm)	SD	Difference	*P*-value
0.017″ × 0.025″ NiTi	0	0.0240	0.1509	0.0223	0.0086	0.0017	0.9721
	5	0.7691	0.1116	3.3690	0.6986	−2.6	*P* < 0.001
	10	2.3667	0.3282	6.3923	1.1822	−4.026	*P* < 0.001
	15	3.9772	0.2970	9.6219	1.0599	−5.645	*P* < 0.001
	20	4.9777	0.5854	11.8697	0.7647	−6.892	*P* < 0.001
	25	6.8471	0.4603	14.9773	0.6659	−8.13	*P* < 0.001
	30	9.8315	0.8743	16.3156	1.1890	−7	*P* < 0.001
	35	13.2189	0.4967	18.4722	1.2781	−5.93	*P* < 0.001
	40	16.6119	1.3309	20.8641	1.3464	−4.252	*P* < 0.001

0.017″ × 0.025″ TMA	0	0.0252	0.0171	0.0295	0.0127	−0.0043	0.5320
	5	0.9051	0.3383	7.0248	0.2327	−6.12	*P* < 0.001
	10	2.6779	0.2815	12.5870	1.8722	−9.909	*P* < 0.001
	15	4.2503	0.6792	18.0033	1.5760	−13.75	*P* < 0.001
	20	8.9140	0.7907	22.8745	3.7282	−13.96	*P* < 0.001
	25	14.1487	1.0334	27.4326	3.3664	−13.28	*P* < 0.001
	30	20.7365	1.7962	32.3051	3.1005	−11.57	*P* < 0.001
	35	27.5439	1.8361	36.4577	3.1192	−8.914	*P* < 0.001
	40	34.2285	1.2383	39.8239	2.7026	−5.595	*P* < 0.001

0.017″ × 0.025″ SS	0	0.02949	0.0176	0.0210	0.0117	0.0085	0.2220
	5	3.2254	0.2472	8.6561	2.6382	−5.431	*P* < 0.001
	10	5.6105	0.7160	18.2694	3.8456	−12.66	*P* < 0.001
	15	8.4498	1.1519	28.6964	3.6621	−20.25	*P* < 0.001
	20	10.5645	1.1355	37.1495	6.1583	−26.59	*P* < 0.001
	25	18.9973	2.9672	45.1034	3.4185	−26.11	*P* < 0.001
	30	29.9413	2.7820	50.7229	3.0109	−20.78	*P* < 0.001
	35	39.9283	3.4029	54.7292	4.8217	−14.8	*P* < 0.001
	40	52.0167	3.7036	58.3516	5.6925	−6.335	*P* < 0.001

0.019″ × 0.025″NiTi	0	0.0385	0.1036	0.0207	0.0088	0.01781	0.5946
	5	1.4661	0.2094	5.4962	1.2149	−4.03	*P* < 0.001
	10	3.1848	0.7159	10.6272	2.9416	−7.442	*P* < 0.001
	15	6.6366	0.5338	15.7175	2.5917	−9.081	*P* < 0.001
	20	11.8557	1.2036	19.5670	3.3616	−7.711	*P* < 0.001
	25	18.1490	1.8227	23.4209	3.5167	−5.272	*P* < 0.001
	30	23.8320	2.9847	27.3286	3.9960	−3.497	0.040
	35	29.1301	3.3593	29.9234	4.8372	−0.7933	0.6752
	40	36.3693	4.6951	33.2707	6.0261	3.099	0.2159

0.019″ × 0.025″ TMA	0	0.0216	0.0132	0.0193	0.0058	0.0023	0.6162
	5	1.7708	0.2226	7.5271	1.7064	−5.756	*P* < 0.001
	10	3.0757	0.2673	13.5094	3.7258	−10.43	*P* < 0.001
	15	7.3887	0.8049	19.8951	5.1686	−12.51	*P* < 0.001
	20	14.9515	1.2012	26.3241	6.1455	−11.37	*P* < 0.001
	25	22.5115	2.3698	31.15591	4.4792	−8.644	*P* < 0.001
	30	30.4845	2.8534	35.62501	3.9194	−5.141	*P* < 0.001
	35	38.2896	2.8789	40.81511	4.4903	−2.526	0.1516
	40	45.7749	2.6586	45.61601	6.5825	0.1588	0.9444

0.019″ × 0.025″ SS	0	0.0185	0.0059	0.0210	0.0134	−0.002	0.5971
	5	3.5466	0.6463	11.0866	1.6574	−7.54	*P* < 0.001
	10	6.6432	1.1633	21.8575	2.2751	−15.21	*P* < 0.001
	15	11.8049	1.9731	32.6077	5.2439	−20.8	*P* < 0.001
	20	24.2718	3.0400	43.7153	4.2239	−19.44	*P* < 0.001
	25	39.9968	2.2740	50.3080	5.3314	−10.31	*P* < 0.001
	30	53.7625	3.1674	55.2497	7.0366	−1.487	0.5498
	35	66.6219	3.3697	57.9423	7.1362	8.68	0.0027
	40	79.2685	4.1318	61.9520	10.5869	17.32	*P* < 0.001

**Table 3:** Comparison of the linear equations, moment, and angle in each group for different bracket types.

Archwire	Conventional bracket		New ribbon bracket		*P*-value
	Linear equation	*R* Square	Linear equation	*R* square	
0.017 × 0.025 inch NiTi	*Y* = 0.7098 ^*∗*^ *X* − 11.78	0.9939	*Y* = 0.5198 ^*∗*^ *X* + 1.126	0.9905	<0.0001
0.017 × 0.025 inch TMA	*Y* = 1.344 ^*∗*^ *X* − 19.6	0.9988	*Y* = 0.9872 ^*∗*^ *X* + 2.448	0.9952	<0.0001
0.017 × 0.025 inch SS	*Y* = 2.251 ^*∗*^ *X* − 38.01	0.9973	*Y* = 1.817 ^*∗*^ *X* + 0.3687	0.9986	<0.0001
0.019 × 0.025 inch NiTi	*Y* = 1.3 ^*∗*^ *X* − 15.73	0.9923	*Y* = 0.8215 ^*∗*^ *X* + 2.13	0.9898	<0.0001
0.019 × 0.025 inch TMA	*Y* = 1.482 ^*∗*^ *X* − 13.8	0.9984	*Y* = 1.118 ^*∗*^ *X* + 2.321	0.9937	<0.0001
0.019 × 0.025 inch SS	*Y* = 2.414 ^*∗*^ *X* − 17.96	0.9970	*Y* = 2.148 ^*∗*^ *X* + 0.1539	0.9990	<0.0001

**Table 4:** Comparison of the moment and angle of rotation to reach 5–20 Nmm for two different bracket types.

	Conventional bracket		New ribbon bracket	
	5°Nmm	20°Nmm	5°Nmm	20°Nmm
0.017 × 0.025 inch NiTi	20	NA	9	39
0.017 × 0.025 inch TMA	17	31	4	18
0.017 × 0.025 inch SS	10	25	3	11
0.019 × 0.025 inch NiTi	14	27	6	20
0.019 × 0.025 inch TMA	14	23	3	15
0.019 × 0.025 inch SS	8	18	3	9

## Data Availability

The datasets used and/or analyzed during the current study are available from the corresponding author on reasonable request.

## Abbreviations

OTS: Orthodontic torque simulator
NiTi: Nickel-titanium
TMA: Titanium molybdenum alloy
SS: Stainless steel.
